# The GSA NIA R13 Diversity Mentoring and Career Development Workshop: what did we learn after 3 years?

**DOI:** 10.1093/geroni/igab046.909

**Published:** 2021-12-17

**Authors:** Patricia D'Antonio, Keith Whitfield, Patricia Heyn

**Affiliations:** 1 The Gerontological Society of America, Washington, District of Columbia, United States; 2 University of Nevada Las Vegas, Las Vegas, Nevada, United States; 3 University of Colorado Anschutz Medical Campus, Arvada, CO, Colorado, United States

## Abstract

The goal of the GSA NIA R13 Diversity Mentoring and Career Development Technical Assistance Workshop (GSA Diversity TAW) is to support, promote, and advance the training of diverse students in aging research. The program’s main aim is to increase the number of early career scientists who are historically underrepresented in gerontological research. Thus, from 2018 to 2020, more than 60 trainees and 16 faculty from diverse backgrounds participated in this unique gerontological training that included peer mentoring opportunities and engagements at the GSA Annual Meeting. The workshop curriculum included scientific presentations, networking, NIH grant preparation, career planning, and effective professional communication. Trainees and faculty were involved in the evaluation of the workshop, which included electronic surveys and focus groups that informed the design and curriculum of subsequent workshops. This paper will discuss the curricular design and objectives of the GSA Diversity TAW and present a summary of the trainees’ feedback results about the program and the iterative changes made based on that data.

